# Improvement in dielectric and mechanical performance of CaCu_3.1_Ti_4_O_12.1 _by addition of Al_2_O_3 _nanoparticles

**DOI:** 10.1186/1556-276X-7-68

**Published:** 2012-01-05

**Authors:** Chompoonuch Puchmark, Gobwute Rujijanagul

**Affiliations:** 1 Department of Physics, Faculty of Science, Naresuan University, Phitsanulok, 65000, Thailand; 2 Department of Physics and Materials Science, Faculty of Science, Chiang Mai University, Chiang Mai, 50200, Thailand

**Keywords:** nanocomposites, dielectric properties, microstructure, mechanical property

## Abstract

The properties of CaCu_3.1_Ti_4_O_12.1 _[CC3.1TO] ceramics with the addition of Al_2_O_3 _nanoparticles, prepared via a solid-state reaction technique, were investigated. The nanoparticle additive was found to inhibit grain growth with the average grain size decreasing from approximately 7.5 μm for CC3.1TO to approximately 2.0 μm for the unmodified samples, while the Knoop hardness value was found to improve with a maximum value of 9.8 GPa for the 1 vol.% Al_2_O_3 _sample. A very high dielectric constant > 60,000 with a low loss tangent (approximately 0.09) was observed for the 0.5 vol.% Al_2_O_3 _sample at 1 kHz and at room temperature. These data suggest that nanocomposites have a great potential for dielectric applications.

## Background

CaCu_3_Ti_4_O_12 _[CCTO] is an interesting dielectric material which exhibits a high dielectric constant over 10,000 at room temperature and shows temperature independence over the temperature range from approximately 100 to 400 K [1-3]. Since the discovery of this material by Subramanian et al. [1], CCTO has been widely studied to further understand and improve its properties. The CCTO crystal has a cubic symmetry with an Im3 space group. In the CCTO lattice, the TiO_6 _octahedra are tilted which results in a doubling of the perovskite-like structure, involved in the planar square arrangement of the oxygen around the copper ions [4]. The CCTO ceramics exhibit an electrically heterogeneous structure involving mobile-charged species in terms of the Maxwell-Wagner relaxation [5]. Internal interfaces in the polycrystalline CCTO give rise to the polarization in the insulating grain boundary and at the semiconducting grains which is well explained by the internal barrier layer capacitor [IBLC] model [6,7]. To improve the dielectric properties further, many cations have been introduced into CCTO, including Co, Zr, Fe, Sc, and Nb on the B site and substitution of La and Eu at the A site [4,8-12]. Although some of these additives resulted in a reduction of the loss tangent, most additives also reduced the dielectric constant. Fang et al. proposed that Cu stoichiometry can affect the electrical properties of the CCTO ceramics, [13] while Kwon et al. reported that both Cu- and Ti-deficient CCTO presented a higher dielectric constant than undoped CCTO [14]. Recently, many authors have reported on the properties of composites between CCTO and other materials such as BaTiO_3_, SrTiO_3_, ZnNb_2_O_6_, and polystyrene [15-17]. However, the properties of composites formed by adding nanocomposites to CCTO have still not been widely investigated. In the present work, a new nanocomposite system between CCTO (with non-stoichiometric composition) and Al_2_O_3 _nanoparticles was fabricated. We demonstrate that the dielectric behavior of the composites can be significantly improved by the addition of these nanoparticles. Some other properties of the nanocomposites were also investigated and reported.

### Experimental procedure

It has been proposed that Cu stoichiometry is related to the dielectric response [13,14]. Fang et al. [13] reported that Cu-excessive CCTO samples showed improved densification and dielectric behaviors. In the present work, Cu-excessive CCTO ceramics in a composition of CaCu_3.1_Ti_4_O_12.1 _[CC3.1TO] were fabricated. Our studies indicate that this composition exhibited a good densification and dielectric response (data not shown). The samples were fabricated using the solid-state mixed oxide method. Reagent grade CaCO_3_, CuO, and TiO_2 _powders were used as starting materials. The mixture of these powders was ground for 24 h in ethanol using zirconia grinding media. The suspension was then dried and subsequently calcined at 900°C for 8 h with a heating rate of 5°C/min. The calcined CC3.1TO powders were mixed with (0.5, 1, and 2 vol.%) Al_2_O_3 _nanoparticles (40 nm average particle size) and 1% polyvinyl alcohol [PVA] binder and were ball-milled in ethanol for 12 h using the same method as mentioned earlier. The slurry was then dried and sieved to a fine powder. The mixed powders were uniaxially pressed into pellets at a pressure of 60 MPa. The PVA binder was burnt out at 550°C with a heating rate of 1°C/min. Finally, the pellets were sintered at 1,025°C for 6 h with a heating rate of 5°C/min. The sintered pellets were investigated for phase formation by X-ray diffraction [XRD]. Density of the sintered samples was measured using the Archimedes method with distilled water as the fluid medium. The microstructures of the sintered samples were characterized using a scanning electron microscope [SEM], and the average grain size was determined using the linear intercept method. For the electrical measurement, silver paste was applied to both sides of the circular faces of the ceramics, then dried at 600°C for 15 min, and cooled naturally to room temperature. The dielectric constant and dielectric loss were then measured using a LCZ meter. The mechanical properties (hardness) of various sintered samples were studied using a Knoop microhardness tester. Indentations were applied to the polished surfaces with 0.3- and 0.5-kg loads and with an indentation period of 15 s.

## Results and discussion

### Phase formation

**Figure 1:**
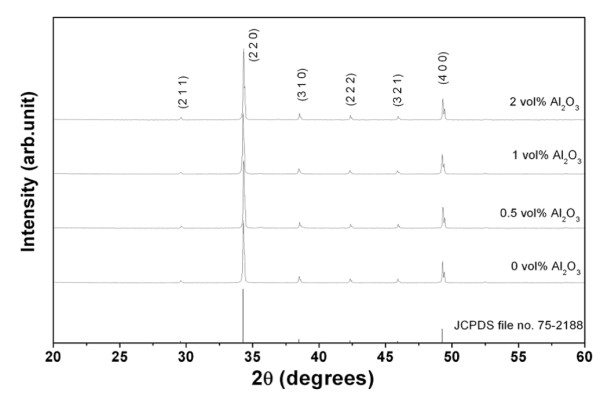
**XRD patterns of the surfaces of the CC3.1TO and CC3.1TO-Al_2_O_3 _pellets**.

The XRD results for the sintered ceramics containing up to 2 vol.% Al_2_O_3 _are illustrated in Figure 1. All of the patterns were similar to the unmodified CCTO diffraction peaks and were consistent with the results reported previously [18]. The peaks of the second phases such as Cu_2_O and CuO could not be observed in the XRD patterns [14]. Further, no peak was observed for the Al_2_O_3 _phase in any of the XRD patterns. This may be due to the amount of Al_2_O_3 _additive which was too little to be detected at the sensitivity level of the XRD instrument.

### Densification, microstructure, and hardness behavior

**Figure 2:**
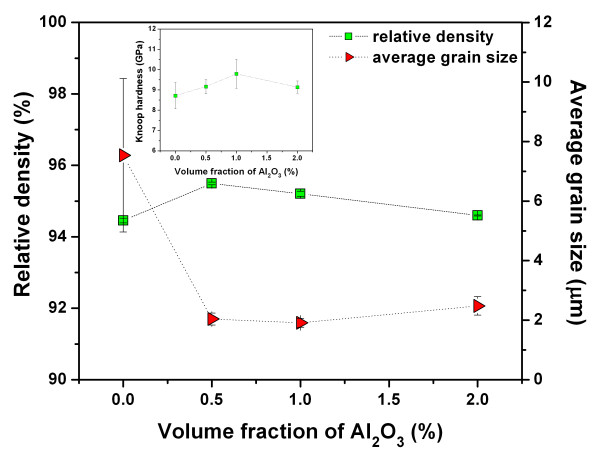
**Density and average grain size as Al_2_O_3 _volume fraction function for CC3.1TO and CC3.1TO-Al_2_O_3 _nanocomposites**. Inset shows Knoop hardness value as a function of Al_2_O_3 _content of the samples.

The plot of density as a function of Al_2_O_3 _volume fraction is shown in Figure 2. The density slightly increased with the increasing amounts of Al_2_O_3 _up to 0.5 vol.% and then decreased for the 2 vol.% sample. The reduction in density for the higher Al_2_O_3 _samples suggests that the sintering mechanism of the samples was not complete. To obtain the best densification for compositions > 0.5 vol.% Al_2_O_3_, higher sintering temperatures or longer soaking times would be required.

**Figure 3:**
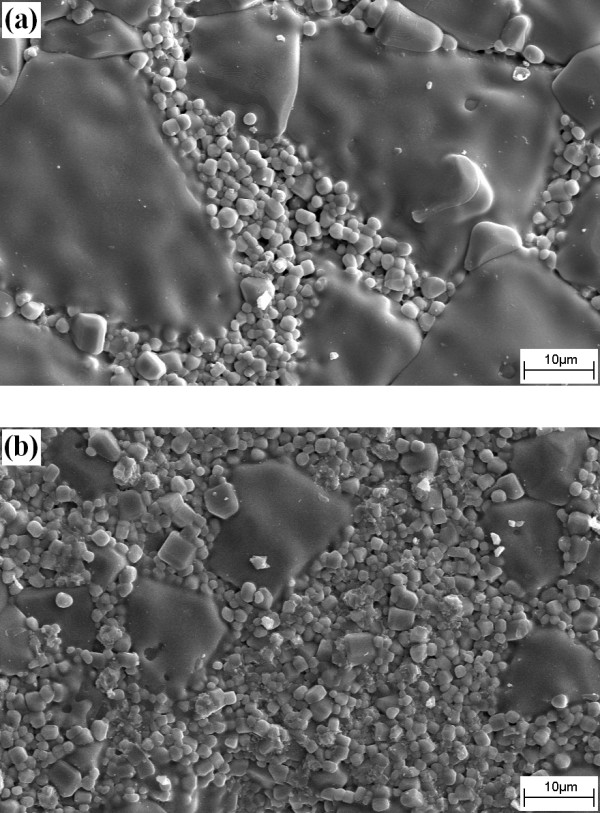
**SEM micrographs of as-sintered surfaces of the CC3.1TO-Al_2_O_3 _nanocomposites**. (**a**) CC3.1TO and (**b**) CC3.1TO and 2 vol.% Al_2_O_3_.

Figure 3 displays the SEM micrographs of the as-sintered surfaces of CC3.1TO-Al_2_O_3 _nanocomposites. An agglomeration of Al_2_O_3 _nanoparticles was not explicitly observed, implying that the processing method produced a reasonably uniform distribution of the nanoparticles in the matrix of the composites. The surfaces of the CC3.1TO samples showed a duplex microstructure consisting of coarse grains (average grain size of approximately 20 μm) and fine grains (average grain size of approximately 1 μm) located around the coarse grains. This characteristic indicates an abnormal grain growth in the microstructure of the samples. The formation of a copper oxide liquid phase (in Cu-excessive CCTO), as suggested by Kim et al. [19], may be the main reason for the formation of abnormal grain growth since the present samples have a Cu-excessive CCTO composition. The liquid phase enhanced nucleation of abnormal grains and the abnormal grains were then formed after sintering. Similar results have been reported previously for Cu-excessive CCTO ceramics [14,20]. The average grain sizes of the coarse grains were found to decrease with the additive (e.g., average grain size of the coarse grains was approximately 3 μm for the 2 vol.% sample; Figure 3b). However, the average grain size of the fine grains remained unchanged for higher Al_2_O_3 _content samples. Overall, the average grain size, calculated from coarse and fine grains, decreased from approximately 7.5 μm for the unmodified sample to approximately 2.0 μm for the 2 vol.% sample (Figure 2). The decrease in the average grain size is most likely caused by the mismatch of the different components. Further, Al_2_O_3 _might segregate to the grain boundaries which could prevent grain boundary movement during the sintering process and, as a result, inhibit grain growth.

The Knoop hardness values of the samples as a function of Al_2_O_3 _content are illustrated in the inset of Figure 2. The Knoop hardness data reveal that the additive improved the hardness values. The maximum hardness value in this work was 9.8 GPa (for the 1 vol.% sample) which is comparable to the value reported by Puchmark et al. for the PZT-Al_2_O_3 _nanocomposites. The improvement in the mechanical properties is most likely due to the nanoparticles reinforcing the grain boundaries and acting as effective pins against microcrack propagation [21]. Moreover, the enhancement of hardness can be related to the reduction in grain size, i.e., small grain size samples gave a higher measured hardness.

### Dielectric properties

**Figure 4:**
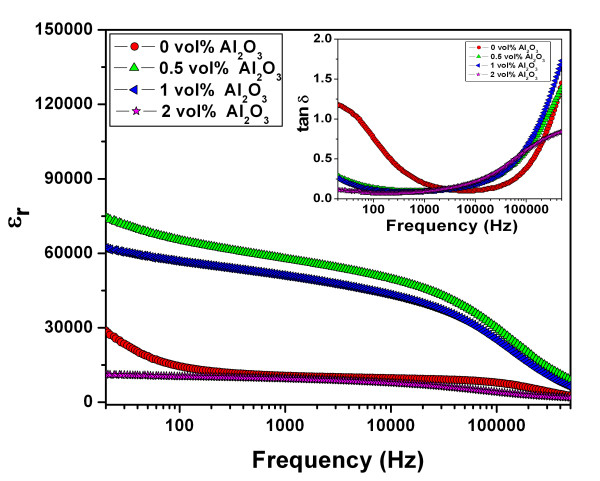
**Dielectric constants versus frequency at room temperature for the CC3.1TO and CC3.1TO-Al_2_O_3 _pellets**. The inset shows the loss tangent versus the frequency of the ceramic pellets at room temperature.

Figure 4 shows the dielectric constants versus the frequency at room temperature for the CC3.1TO and CC3.1TO-Al_2_O_3 _pellets. Compared with the CC3.1TO sample, a significant improvement in the dielectric constant of the CC3.1TO-Al_2_O_3 _samples was observed. For the CC3.1TO sample, the dielectric constant was 11,000 (measured at 1 kHz and at room temperature) which is close to the values reported previously [1,2]. The CC3.1TO sample also exhibited nearly dielectric-frequency independence over the frequency range of 0.1 to 500 kHz. Further, the dielectric constant increased reaching a value > 60,000 at 1 kHz for the 0.5 vol.% sample then decreased for further increases in the Al_2_O_3 _content. For the 2 vol.% sample, however, the dielectric-frequency curve showed a weak frequency dispersion of the dielectric constant. The reduction in the dielectric constant for the samples which were doped with more than 0.5 vol.% Al_2_O_3 _may be due to the fact that composites with higher additive amounts (Al_2_O_3 _> 0.5 vol.%) also had higher structural heterogeneity. Moreover, the formation of an impurity phase may have caused a reaction between Al_2_O_3 _and CC3.1TO which could not be detected using the XRD technique [22], but it might also have contributed to the reduction in the dielectric constant. Plots of the loss tangent versus the frequency of the CC3.1TO and CC3.1TO-Al_2_O_3 _pellets at room temperature are presented in the inset of Figure 4. The loss tangent-frequency curve of the CC3.1TO ceramic exhibited a weak frequency dispersion for a narrow frequency range (1 to 50 kHz). However, after adding the additive, the loss tangent significantly decreased at low frequencies (< 1 kHz) which resulted in a wider range of frequency stability (10 Hz to 10 kHz). Further, the loss tangent decreased from 0.21 for the CC3.1TO ceramics to 0.09 for the 0.5 vol.% sample (at 1 kHz). A further slight decrease in the loss tangent was observed for additional additive amounts. The decrease in loss tangent shows a reduced conductivity of the CC3.1TO-Al_2_O_3 _samples. This result could be related to changes in the transport behavior due to an increase in resistivity at the grain boundaries where the additive nanoparticles predominantly segregated.

**Figure 5:**
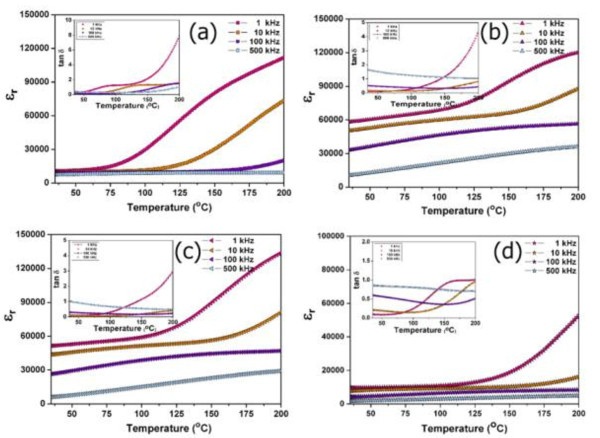
**Temperature dependence of the dielectric dispersion at various frequencies**. (**a**) CC3.1TO, (**b**) CC3.1TO with 0.5 vol.% Al_2_O_3_, (**c**) CC3.1TO with 1 vol.% Al_2_O_3_, and (**d**) CC3.1TO with 2 vol.% Al_2_O_3_.

Figure 5 shows the dielectric constant values as a function of temperature at various frequencies for the CC3.1TO and CC3.1TO-Al_2_O_3 _pellets. The CC3.1TO sample showed a high dielectric constant (*ε_r _*approximately 10,000) with temperature and frequency stability from room temperature to 60°C. After adding the additive, however, a significant improvement in the dielectric behavior was observed. The 0.5 vol.% sample showed a very high dielectric constant > 60,000 (at 1 kHz) which was nearly temperature-independent for the temperature range of approximately 35°C to 110°C. Compared to the CC3.1TO sample, this sample also displayed a pronounced frequency dependence of the dielectric constant especially for temperatures < 125°C. Moreover, the dielectric-temperature curve of the 0.5 vol.% presented a broad flat curve at high frequencies (> 10 kHz). For higher additive amounts (Al_2_O_3 _> 0.5 vol.%), the dielectric constant decreased with the increasing additives. Further, the dielectric frequency dispersion for the Al_2_O_3 _nanoparticle sample with 2 vol.% was not as strong for temperatures < 112°C. The loss tangent values as a function of temperature at various frequencies for the samples are illustrated in the insets of Figure 5. For the CC3.1TO sample, the loss tangent value was 0.21 and was stable with temperature as well as frequency from room temperature to approximately 41°C. The 0.5 vol.% sample had a loss tangent value lower than 0.10 for room temperature to approximately 50°C. From the (IBLC) model, the apparent dielectric constant ($\varepsilon_{r}^{\prime }$) can be related to the microstructure parameters by the formula [23]:

(1) $$\varepsilon_{r}^{\prime }=\varepsilon_{\text{gb}}\;\left(\frac{d}{t}\right),$$

where *d *is the grain size, *t *is the thickness of the grain boundary (barrier width), and *ε*_gb _is the internal dielectric constant of the barrier material. Since the grain size of the present samples decreased with the increasing additive, Equation 1 predicts that the higher dielectric constant for the 0.5 vol.% sample is not related to the grain size, but it may be connected to a change in the grain boundary characteristics such as *ε*_gb _and *t *after adding the additive. The reason for the change of grain boundary characteristic is still unclear, but it is possible that the Al_2_O_3 _nanoparticles had a reaction with the matrix of the CC3.1TO, and as a result, the formation of Al-metal oxide phases at the grain boundary produced other products in small amounts which could not be detect by XRD [22]. However, the higher density for the 0.5 vol.% sample can be explained by the observed higher dielectric constant in the present work.

## Conclusions

CC3.1TO-Al_2_O_3 _nanocomposites were fabricated for the first time. The samples were prepared using a solid-state reaction. The CC3.1TO ceramics showed a duplex microstructure, consisting of coarse and fine grains, while the nanocomposites showed mainly fine grains in their microstructure due to the fact that the additive inhibited grain growth. The additive also enhanced the hardness value especially for the 1 vol.% sample. However, the CC3.1TO and 0.5 vol.% Al_2_O_3 _showed a high dielectric constant with a strong dielectric frequency dispersion especially at low temperatures and also had a lowered loss tangent value, as compared with other samples. These results indicate that the addition of nanoparticles may be an alternative method to improve the dielectric behavior in some other giant dielectric materials.

## Competing interests

The authors declare that they have no competing interests.

## Authors' contributions

CP carried out the fabrication of CC3.1TO, XRD characterization, SEM characterization, density measurement, grain size measurement, dielectric properties measurement, and Knoop hardness measurement. GR designed the whole experimental procedure and related analyses. All authors read and approved the final manuscript.
